# Noise filter using a periodic system of dual Helmholtz resonators

**DOI:** 10.1038/s41598-024-74799-2

**Published:** 2024-10-23

**Authors:** Mohamed El Malki, Ali Khettabi, Mohammed Sallah, Zaky A. Zaky

**Affiliations:** 1 Laboratory of Materials, Waves, Energy and Environment, Department of Physics, Faculty of Sciences, Mohammed First University, Oujda, 60000 Morocco; 2 Department of Physics, College of Sciences, University of Bisha, P.O. Box 344, Bisha, 61922 Saudi Arabia; 3 TH-PPM Group, Physics Department, Faculty of Science, Beni-Suef University, Beni Suef, 62514 Egypt; 4 Academy of Scientific Research and Technology (ASRT), Cairo, Egypt; 5 Frank Laboratory of Neutron Physics, Joint Institute for Nuclear Research, Dubna, 141980 Russia

**Keywords:** Noise reduction, Periodic structure, Dual Helmholtz resonator, Transmission, Defect mode, Computational science, Mechanical properties, Computational methods, Mechanical engineering, Fluidics

## Abstract

This study investigates noise reduction using a periodic arrangement of dual Helmholtz resonators and explores the introduction of defects within this periodic structure. The transfer matrix method was employed to carry out theoretical research. The computations of the interface response function approach results are verified, and consistent outcomes are demonstrated. The simulation results highlight the distinctive dual resonance frequencies of dual Helmholtz resonators. By differentiating dual Helmholtz resonators from traditional Helmholtz resonators, prospective applications for low-frequency noise reduction are envisioned. In this contribution, introducing defects in the middle of perfect dual Helmholtz resonators adds more value to the acoustic filter. In particular, the first neck and cavity of the defective dual Helmholtz resonator. This study shows that introducing a 2D-defect into identical dual Helmholtz resonators can improve the transmission of defect modes by taking advantage of the advantageous interaction of the resonant modes. In such arrangements, the entire structure functioned as a potent selective filter.

**Supplementary Information:**

The online version contains supplementary material available at 10.1038/s41598-024-74799-2.

## Introduction

Designs that are repeated in space or time at regular intervals are referred to as periodic structures^1^. Periodic structures represent a novel and efficient method for attenuating noise and vibrations in acoustics^2^. A significant portion of the theory of periodic structures is based on solid-state physics^3^. The solid-state physical signature has a band structure. However, the same phenomenon has also been observed in other fields. The fact that these structures can only support waves in particular bands is a characteristic that sets them apart^4^. Acoustic filters from periodic structures are commonly utilized^5,6^. One strategy for reducing noise is by changing the waveguide’s cross-sectional area. Acoustic filters have been extensively studied over the past century. These resonators can be selective, high-pass, or low-pass filters^7^. By incorporating scatterers or local resonators (LRs) into a base material, a locally resonant bandgap (LRBG) can be produced^8^. These LR can trap or scatter waves in a way that results in LRBG, and they have their own inherent frequencies. LRBG is formed by the interaction between the LR modes of the scatterers. The interfering of resonant frequencies of LRs creates localized vibration patterns that disrupt the movement of waves. The LRBG depends on the resonance characteristics of the local inclusions. The frequency range of the LRBG is affected by the characteristics of the LRs, like their size, shape, and base material.

Helmholtz resonators (HRs) are efficient noise reduction filters^9,10^. A single HR typically comprises a cavity and a neck organized in a cascade. The purpose of the HRs’ resonance peak is to control noise in a specific frequency band. Attenuation over a wide frequency range is accomplished by periodic HR configurations^11,12^. Recently, studies have attempted HRs in series and parallel configurations in ventilation duct systems to improve the attenuation offered by these resonators^10,13^. Research on the transmission loss (TL) of series configurations of HRs examines various factors, such as the spacing between resonators, their position or orientation, and the dimensions of the HRs^14–16^. These configurations have demonstrated the potential to significantly increase attenuation levels. Recently, periodic structures consisting of dual Helmholtz resonators (DHRs) have attracted considerable research interest owing to their ability to provide substantial attenuation compared to single-HR configurations^17^. Single HR has also been explored as a dissipative silencer that incorporates simple absorbers of fiber and membrane materials^18^. Interestingly, DHRs, which are composed of two HRs in a cascade, have two resonant frequencies that change depending on the size of the resonators^18,19^. DHRs with square ducts exhibit certain acoustic properties^20^, and more studies have been conducted to experimentally assess how the size and layout affect the overall efficacy of noise attenuation^21^.

One of the main ways to effectively use band gaps is to introduce defects to localize modes within the band gaps^22–30^. The geometrical characteristics of the defect, such as the boundary conditions and number of defects incorporated into the structure, significantly impact these modes. The effects of defects in HRs embedded in periodic structures have been studied using the transfer matrix method (TMM)^31^. The spacing between adjacent resonators and the dimensions of the resonators characterize these defects. Defects can selectively influence the modes within the LRBG. Studies on acoustic wave propagation via a sequence of HRs clear that the acoustic structures are unlike traditional elastic examples, especially when defects are present^32^.

This study analyzes periodic DHRs, which are commonly employed for passive noise attenuation in ducts. HRs are economical solutions widely used at low frequencies to enhance sound absorption and insulation in various applications^33–37^. HRs are sound amplification devices in resonance. Perfect DHRs have been the subject of much research^38–41^, whereas defects in these resonators have received less attention. The objective was to examine the acoustic behavior of the DHRs without mean flow and dissipative effects. In addition, we evaluated the impact of the defects included in the periodic structure. A theoretical framework for periodic DHRs, which is then validated analytically by applying the interface response function approach (IRF) and the TMM, is investigated. Our results are robust and in agreement with the experimental observations. Mutual interaction effects amplify localized modes inside band gaps and attenuate sound within passbands, which are studied by introducing two flaws into a single resonator.

## Theoretical model

### Transfer matrix analysis

**Fig. 1:**
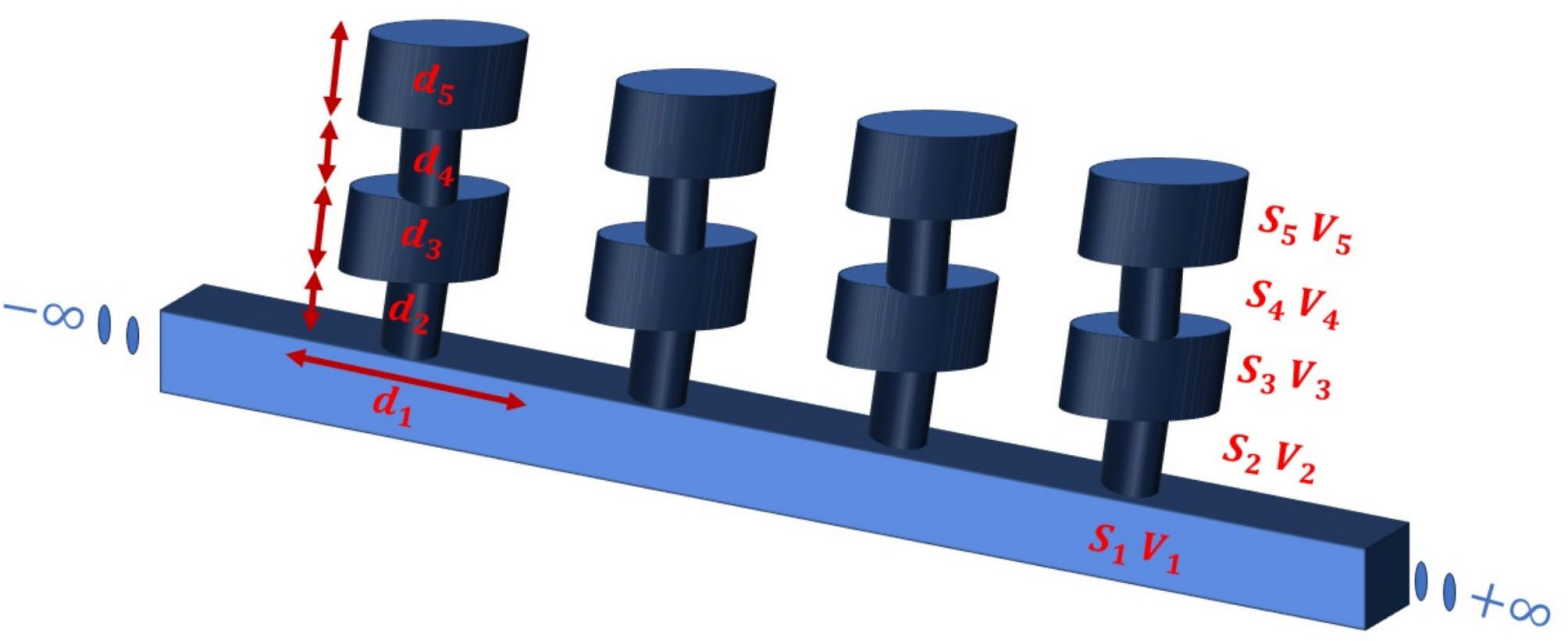
Diagram of an infinite periodic structure formed by DHRs grafted periodically over a given period N.

Figure 1 represents the infinite structure where two HRs are joined to form the entire system. Each cell is composed of one dual HR connected to the main guide, and both contain air. A DHR is formed by a pair consisting of a rigid-walled cavity (neck-cavity-neck-cavity) of volume $\:{V}_{i}$, cross-sections $\:{s}_{i}\:$ and lengths $\:{d}_{i}(i=2,\:3,\:4,\:5)$. For the frequency of interest, we assume that $\:\lambda\:\gg\:{d}_{i},{V}_{i}^{\frac{1}{3}},{s}_{i}^{\frac{1}{2}}\:(i=1,\:2,\:3,\:4,\:5)$^7^. In this investigation, the effects of dissipation will not be considered for simplicity.

**Fig. 2:**
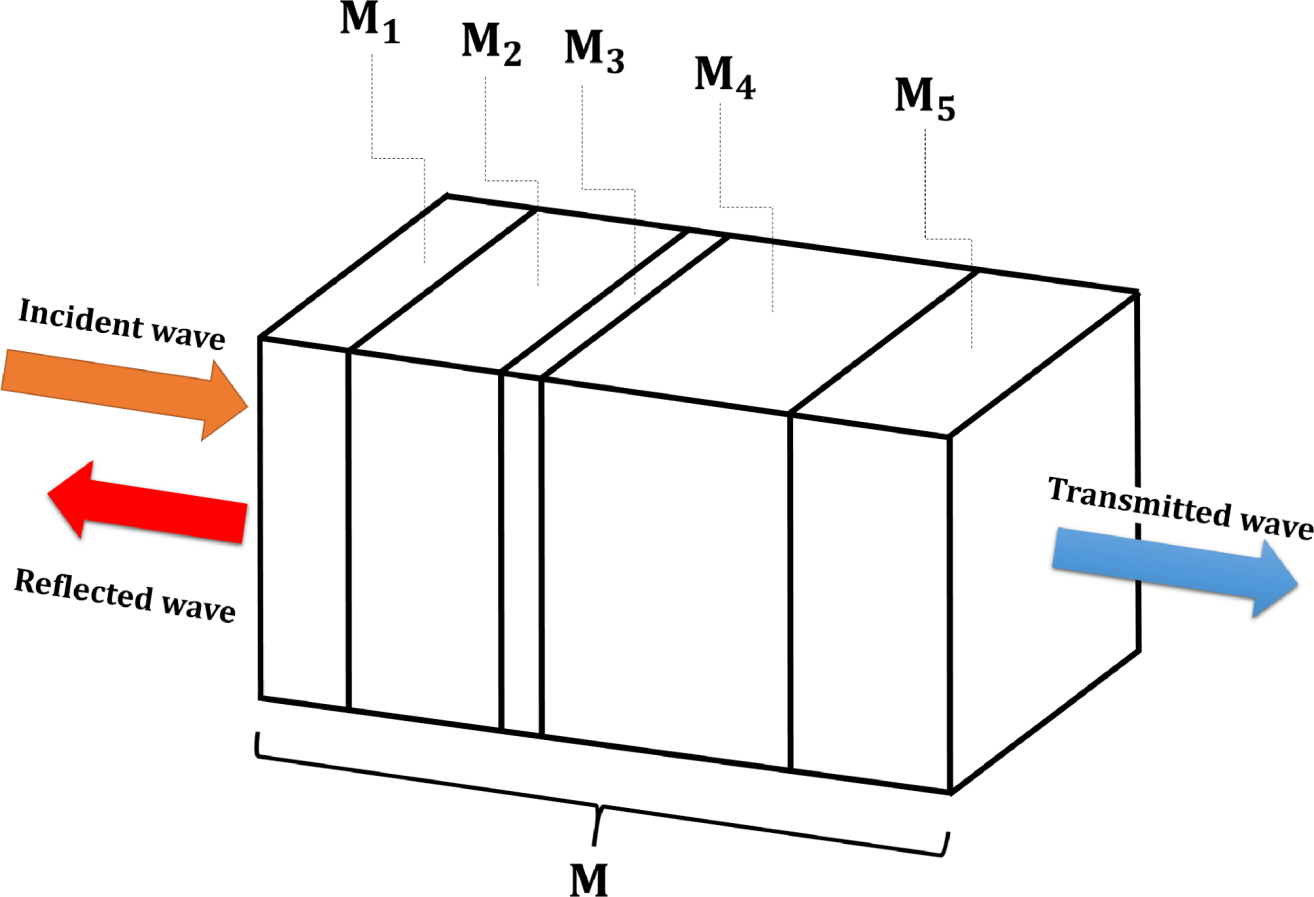
TMM modeling diagram of a simple multilayer system. $\:{M}_{i}\:(i\:=\:1,\:\dots\:,\:5)\:$ is the transfer matrix of layer $\:i\:\text{while}\:M$ being the transfer matrix of the complete system ($\:M=\prod\:_{i=1}^{5}{M}_{i}$).

By analyzing the transmission of an acoustic wave across a multilayer system of multiple media, the TMM can be considered as an efficient analytical calculation tool. A straightforward example of a multilayer structure is represented in Fig. 2. The simple division of a scheme into a certain number of segments or elements, each are represented by a transfer matrix, forms the basis of the TMM. A global matrix (M), which is provided by the product of the set of matrices of each cell, can be inferred by maintaining the pressure and acoustic velocity continuity between each layer. The dominant sound pressure in the guide is defined as follows:

The sound pressure prevailing in the guide is defined by:

1 $$\:p(z,t)=P\left(z\right){\text{e}}^{-\text{j}\omega\:t},$$

where,

2 $$\:P\left(z\right)=A{\text{e}}^{+\text{j}\text{k}\text{z}}+\:B{\text{e}}^{-\text{j}\text{k}\text{z}},$$

with $\:\text{k}=\omega\:/{c}_{0}$, $\:{c}_{0}=343\:m/s,$ and $\:\omega\:=2\pi\:f$, $\:f$ is the source frequency. The acoustic velocity can be obtained using Euler’s equation:

3 $$\:\frac{\partial\:p}{\partial\:z}=-{\rho\:}_{0}\frac{\partial\:v}{\partial\:t},$$

where $\:{\rho\:}_{0}\:$ is the density of air ($\:{\rho\:}_{0}=1.2\:\text{k}\text{g}/{\text{m}}^{3}$). The volume velocity $\:u$ can be deduced using the following equation:

4 $$\:\overrightarrow{u}=s\overrightarrow{v}.$$

**Fig. 3:**
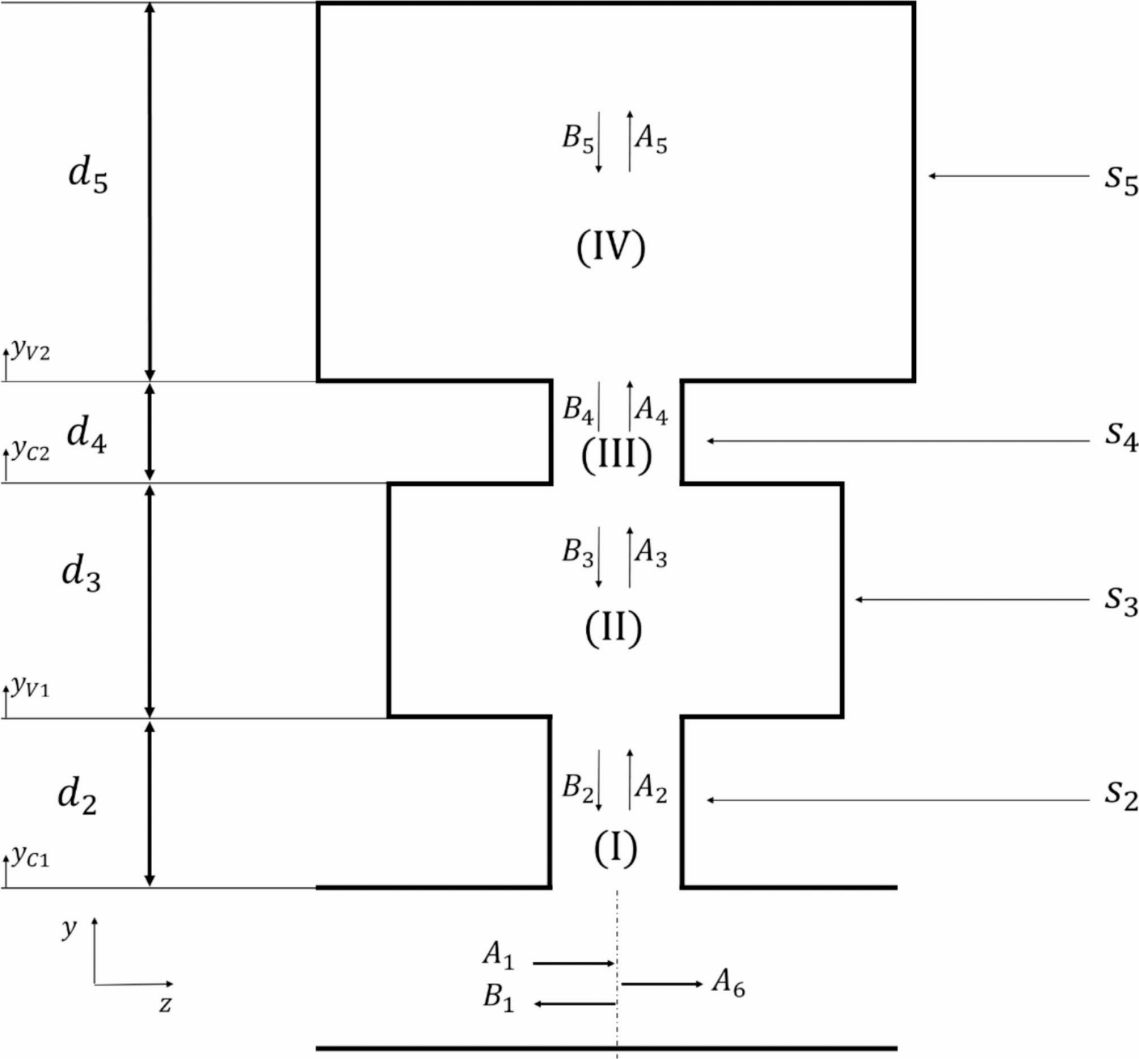
Transmission of acoustic waves in a DHR (one-dimensional model).

Now, let us consider the periodic structure created by the DHR (Fig. 1). The following equation provides the pressure and acoustic flow continuity at the interface between the main duct and the first neck of the DHR (Fig. 3):

5 $$\:{A}_{1}+{B}_{1}={A}_{2}+{B}_{2}={A}_{6},$$

6 $$\:{s}_{1}\left[{A}_{1}-{B}_{1}\right]={s}_{2}\left[{A}_{2}-{B}_{2}\right]+{s}_{5}{A}_{5},$$

where $\:{s}_{1}$ and $\:{s}_{2}$ are the cross-sections of the main guide and the first neck of the DHR, $\:{s}_{5}$ is that of the 2nd cavity. The continuity of sound pressure between domains (I), (II), (III) and (IV) are given by:

7 $$\:{P}_{2}\left({y}_{C1}={d}_{2}\right)={P}_{3}\left({y}_{V1}=0\right).$$

8 $$\:{P}_{3}\left({y}_{V1}={d}_{3}\right)={P}_{4}\left({y}_{C2}=0\right),$$

9 $$\:{P}_{4}\left({y}_{C2}={d}_{4}\right)={P}_{5}\left({y}_{V2}=0\right),$$

where $\:{y}_{Ci}(i=1,\:2)$ is the characteristic acoustic admittance of the DHR necks while $\:{y}_{Vi}$ are those of its cavities. The continuity of the acoustic flow is defined as:

10 $$\:{s}_{2}{u}_{2}\left({y}_{C1}={d}_{2}\right)={s}_{3}{u}_{3}\left({y}_{V1}=0\right){\text{at the junction}}\,\, {y}_{C1}={d}_{2}\:\text{o}\text{r}\:{y}_{V1}=0,$$

11 $$\:{s}_{3}{u}_{3}\left({y}_{V1}={d}_{3}\right)={s}_{4}{u}_{4}\left({y}_{C2}=0\right)\:{\text{at the junction}}\,\, {y}_{V1}={d}_{3}\:\text{o}\text{r}\:{y}_{C2}=0,$$

12 $$\:{s}_{4}{u}_{4}\left({y}_{C2}={d}_{4}\right)={s}_{5}{u}_{5}\left({y}_{V2}=0\right){\text{at the junction}}\,\, {y}_{C2}={d}_{4}\:\text{o}\text{r}\:{y}_{V2}=0.$$

The relationship between the acoustic pressure and flow at the DHR is expressed as:

13 $$\:\left(\begin{array}{c}{P}_{1}\\\:{u}_{1}\end{array}\right)={M}_{\text{D}\text{H}\text{R}}\left(\begin{array}{c}{P}_{5}\\\:{u}_{5}\end{array}\right),\:$$

where $\:{u}_{5}=0$ for $\:{y}_{V2}={d}_{5}$, whereas $\:{M}_{DHR}$ is the transfer matrix of the DHR.

14 $$\begin{aligned}{M}_{\text{DHR}}&=\left(\begin{array}{cc}{A}_{2}&\:{B}_{2}\\\:{C}_{2}&\:{D}_{2}\end{array}\right)\left(\begin{array}{cc}{A}_{3}&\:{B}_{3}\\\:{C}_{3}&\:{D}_{3}\end{array}\right)\left(\begin{array}{cc}{A}_{4}&\:{B}_{4}\\\:{C}_{4}&\:{D}_{4}\end{array}\right)\left(\begin{array}{cc}{A}_{5}&\:{B}_{5}\\\:{C}_{5}&\:{D}_{5}\end{array}\right)\\ &=\left(\begin{array}{cc}{A}_{\text{DHR}}&\:{B}_{\text{DHR}}\\\:{C}_{\text{DHR}}&\:{D}_{\text{DHR}}\end{array}\right).\end{aligned}$$

where

15 $$\begin{aligned} {A}_{\text{D}\text{H}\text{R}}&=cos\left(k{d}_{2}\right)cos\left(k{d}_{3}\right)cos\left(k{d}_{4}\right)cos\left(k{d}_{5}\right)-{z}_{4}{y}_{5}cos\left(k{d}_{2}\right)\\&\quad \times sin\left(k{d}_{3}\right)sin\left(k{d}_{4}\right)sin\left(k{d}_{5}\right)-{z}_{3}{y}_{4}cos\left(k{d}_{2}\right)cos\left(k{d}_{5}\right)\\&\quad \times sin\left(k{d}_{3}\right)sin\left(k{d}_{4}\right)-{z}_{3}{y}_{5}cos\left(k{d}_{2}\right)cos\left(k{d}_{4}\right)sin\left(k{d}_{3}\right)\\&\quad \times sin\left(k{d}_{5}\right)-{z}_{2}{y}_{3}cos\left(k{d}_{4}\right)cos\left(k{d}_{5}\right)sin\left(k{d}_{2}\right)sin\left(k{d}_{3}\right)\\&\quad -{z}_{2}{y}_{4}cos\left(k{d}_{3}\right)cos\left(k{d}_{5}\right)sin\left(k{d}_{2}\right)sin\left(k{d}_{4}\right)+{z}_{2}{z}_{4}{y}_{3}\\&\quad \times {y}_{5}sin\left(k{d}_{2}\right)sin\left(k{d}_{3}\right)sin\left(k{d}_{4}\right)sin\left(k{d}_{5}\right)-{z}_{2}{y}_{5}\\&\quad \times \text{cos}\left(k{d}_{3}\right){cos}\left(k{d}_{4}\right){sin}\left(k{d}_{2}\right){sin}\left(k{d}_{5}\right).\:\end{aligned}$$

16 $$\begin{aligned}{B}_{\text{D}\text{H}\text{R}}&=j{[z}_{5}cos\left(k{d}_{2}\right)cos\left(k{d}_{3}\right)cos\left(k{d}_{4}\right)sin\left(k{d}_{5}\right)+{z}_{4}cos\left(k{d}_{2}\right)\\&\quad \times cos\left(k{d}_{3}\right)sin\left(k{d}_{4}\right)sin\left(k{d}_{5}\right){z}_{3}{y}_{4}{y}_{5}cos\left(k{d}_{2}\right)sin\left(k{d}_{3}\right)\\&\quad \times sin\left(k{d}_{4}\right)sin\left(k{d}_{5}\right)+{z}_{3}cos\left(k{d}_{2}\right)sin\left(k{d}_{3}\right)cos\left(k{d}_{4}\right)\\&\quad \times cos\left(k{d}_{5}\right)-{z}_{2}{y}_{3}{z}_{5}sin\left(k{d}_{2}\right)sin\left(k{d}_{3}\right)cos\left(k{d}_{4}\right)sin\left(k{d}_{5}\right)],\end{aligned}$$

17 $$\begin{aligned}{C}_{\text{D}\text{H}\text{R}}&=j\left[{y}_{2}\text{s}\text{i}\text{n}\left(k{d}_{2}\right)\text{c}\text{o}\text{s}\left(k{d}_{3}\right)\text{c}\text{o}\text{s}\left(k{d}_{4}\right)\text{c}\text{o}\text{s}\left(k{d}_{5}\right)-{z}_{4}{y}_{2}{y}_{5}\text{s}\text{i}\text{n}\left(k{d}_{2}\right)\right.\\&\quad\times cos\left(k{d}_{3}\right)sin\left(k{d}_{4}\right)sin\left(k{d}_{5}\right)-{z}_{3}{y}_{2}{y}_{4}sin\left(k{d}_{2}\right)sin\left(k{d}_{3}\right)\\&\quad\times sin\left({k}_{4}{d}_{4}\right)cos\left(k{d}_{5}\right)-{z}_{3}{y}_{2}{y}_{5}sin\left(k{d}_{2}\right)sin\left(k{d}_{3}\right)cos\left(k{d}_{4}\right)\\&\quad\times sin\left(k{d}_{5}\right)+{y}_{3}cos\left(k{d}_{2}\right)sin\left(k{d}_{3}\right)cos\left(k{d}_{4}\right)cos\left(k{d}_{5}\right)\\&\quad -{z}_{4}{y}_{3}{y}_{5}cos\left(k{d}_{2}\right)sin\left(k{d}_{3}\right)sin\left(k{d}_{4}\right)sin\left(k{d}_{5}\right)\\&\quad +{y}_{4}sin\left(k{d}_{2}\right)cos\left(k{d}_{3}\right)cos\left(k{d}_{4}\right)cos\left(k{d}_{5}\right)\\&\quad +\left.{y}_{5}\text{cos}\left(k{d}_{2}\right)\text{cos}\left(k{d}_{3}\right)\text{cos}\left(k{d}_{4}\right)\text{sin}\left(k{d}_{5}\right)\right],\end{aligned}$$

18 $$\begin{aligned}{D}_{\text{D}\text{H}\text{R}}&=cos\left(k{d}_{2}\right)cos\left(k{d}_{3}\right)cos\left(k{d}_{4}\right)cos\left(k{d}_{5}\right)-{y}_{4}{z}_{5}cos\left(k{d}_{2}\right)\\&\quad\times sin\left(k{d}_{3}\right)sin\left(k{d}_{4}\right)sin\left(k{d}_{5}\right)-{y}_{3}{z}_{4}cos\left(k{d}_{2}\right)cos\left(k{d}_{5}\right)\\&\quad\times sin\left(k{d}_{3}\right)sin\left(k{d}_{4}\right)-{y}_{3}{z}_{5}cos\left(k{d}_{2}\right)cos\left(k{d}_{4}\right)sin\left(k{d}_{3}\right)\\&\quad\times sin\left(k{d}_{5}\right)-{y}_{2}{z}_{3}cos\left(k{d}_{4}\right)cos\left(k{d}_{5}\right)sin\left(k{d}_{2}\right)sin\left(k{d}_{3}\right)\\&\quad +{z}_{2}{z}_{4}{y}_{3}{y}_{5}sin\left(k{d}_{2}\right)sin\left(k{d}_{3}\right)sin\left(k{d}_{4}\right)sin\left(k{d}_{5}\right)-{y}_{2}\\&\quad\times {z}_{4}cos\left(k{d}_{3}\right)cos\left(k{d}_{5}\right)sin\left(k{d}_{2}\right)sin\left(k{d}_{4}\right)-{y}_{2}{z}_{5}cos\left(k{d}_{3}\right)\\&\quad\times \text{cos}\left(k{d}_{4}\right)\text{sin}\left(k{d}_{2}\right)\text{sin}\left(k{d}_{5}\right),\end{aligned}$$

**Fig. 4:**
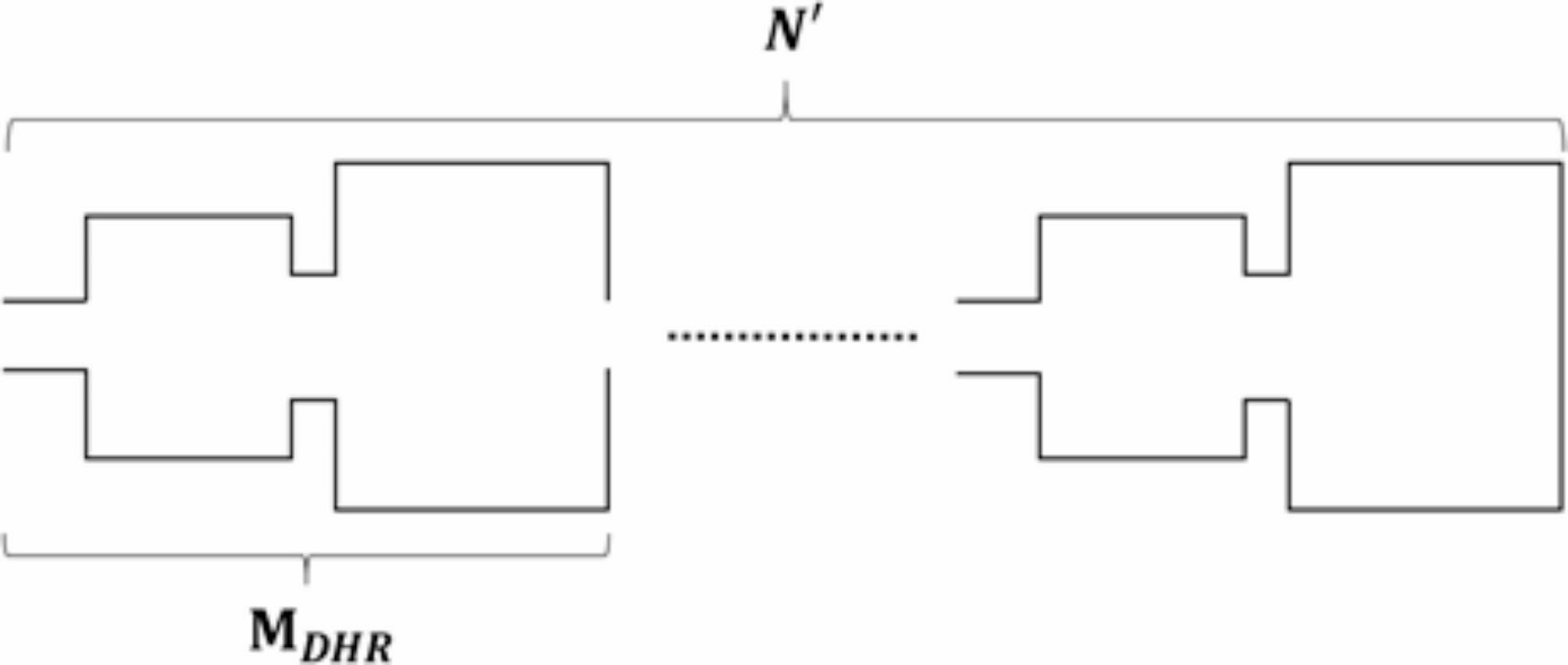
TMM modeling scheme for a system of $N^{\prime}$ cascaded DHR.

The simplicity of the TMM allows us to conclude the general case of N′ DHR connected in a cascade. Clearly, the number of DHR connected in a cascade is proportional to the resonant frequency. Three peaks are produced in a hydraulic system formed by three HRs, which proves our prediction^40^. The TL is studied using the TMM which is based on a distributed parameter model of hydraulic piping^42^. Considering Eq. (14), the transfer matrix of a finite number $N^{\prime}$ of DHR ($\:{M}_{{\text{D}\text{H}\text{R}}_{n}}$) in cascade (Fig. 4) is defined as follows:

19 $$\:{M}_{{\text{DHR}}_{n}}={M}_{\text{DHR}}^{{N}^{{\prime}}}.$$

Using Eq. (13), we have:

20 $$\:\begin{array}{c}{P}_{1}={A}_{\text{DHR}}{P}_{5}\\\:{u}_{1}={C}_{\text{DHR}}{P}_{5}.\end{array}$$

The impedance of the DHR equals:

21 $$\begin{aligned} {z}_{\text{D}\text{H}\text{R}}=\frac{{p}_{1}}{{u}_{1}}=\frac{{A}_{\text{DHR}}}{{C}_{\text{DHR}}}.\end{aligned}$$

If we consider a special case in which $\:{d}_{2}={d}_{4}$ and $\:{d}_{3}={d}_{5}$, the elements of Eq. (21) become:

22a $$\begin{aligned}{C}_{\text{DHR}}&=j\left[2\left({y}_{2}{\text{tan}}\left(k{d}_{2}\right)-{y}_{3}{\text{tan}}\left(k{d}_{3}\right)\right)-\left({y}_{3}+{z}_{2}{y}_{2}^{2}\right){\text{tan}}^{2}\left(k{d}_{2}\right)\right.\\&\quad \times \left.{\text{tan}}\left(k{d}_{3}\right)-\left({y}_{2}+{z}_{2}{y}_{3}^{2}\right){\text{tan}}\left(k{d}_{2}\right){\text{tan}}^{2}\left(k{d}_{3}\right)\right].\end{aligned}$$

22b $$\begin{aligned}{A}_{\text{DHR}}&=1-\left(3{z}_{2}{y}_{3}+{z}_{3}{y}_{2}-{z}_{2}^{2}{y}_{3}^{2}{\text{tan}}\left(k{d}_{2}\right){\text{tan}}\left(k{d}_{3}\right)\right){\text{tan}}\left(k{d}_{2}\right)\\&\quad \times {\text{tan}}\left(k{d}_{3}\right)-{\text{tan}}^{2}\left(k{d}_{2}\right)-{\text{tan}}^{2}\left(k{d}_{3}\right).\end{aligned}$$

When the wavelength of the propagating acoustic wave is substantially larger than the resonator’s size, it can be approximated as $\:\text{t}\text{a}\text{n}\left(k{d}_{i}\right)\approx\:k{d}_{i}\ll\:1\:(i=2-5)$. Hence, a simple form of the acoustic impedance of the DHR is given as:

23 $$\begin{aligned} {z}_{\text{DHR}}=\text{j}\omega\:{\rho\:}_{0}{d}_{2}\frac{({-\omega\:}^{2}/{\omega\:}_{3}^{2}+1)\left({-\omega\:}^{2}/{\omega\:}_{5}^{2}+1\right)-R}{({-\omega\:}^{2}/{\omega\:}_{3}^{2}+1)\left({-\omega\:}^{2}/{\omega\:}_{5}^{2}+R\right)-R};\quad \text{for}\:{d}_{2}={d}_{4}\:\text{and}\:{d}_{3}={d}_{5}\end{aligned}$$

The two resonance Eigen frequencies of the DHR are determined for $\:{z}_{DHR}=0$ as follows:

24 $$\:{\left({\omega\:}^{\pm\:}\right)}^{2}=\frac{1}{2}\left({\omega\:}_{3}^{2}+{\omega\:}_{5}^{2}\pm\:\sqrt{{{\left({\omega\:}_{3}^{2}-{\omega\:}_{5}^{2}\right)}^{\:}}^{2}-4\text{R}{\omega\:}_{3}^{2}{\omega\:}_{5}^{2}}\right),$$

where $\:R=\left({s}_{4}/{s}_{2}\:\right)/\left({d}_{4}/{d}_{2}+{s}_{4}/{s}_{2}\right)$, $\:{\omega\:}_{3}^{\:}=\:\sqrt{{c}_{0}^{2}\left({s}_{2}/{d}_{2}+{s}_{4}/{d}_{4}\right)/{V}_{4}}$, $\:{\omega\:}_{5}^{\:}=\:\sqrt{{c}_{0}^{2}{s}_{4}/{d}_{4}{V}_{4}}$, $\:{V}_{i},\:{d}_{i},$ and $\:{s}_{i}\:(i=2-5)$ are the volumes of DHR elements (necks and cavities), their lengths and cross-sections, respectively. $\:{z}_{\text{D}\text{H}\text{R}}$ in Eq. (21) satisfies Eq. (S6), obtained using the IRF approach (see supplementary material). The TL can be calculated as follows:

25 $$\:\text{T}\text{L}=20\text{l}\text{o}\text{g}\left|1+\frac{1}{2}\frac{{z}_{1}}{{z}_{\text{D}\text{H}\text{R}}}\right|.$$

The transfer matrix of a DHR cell is given as:

26 $$\begin{aligned}{M}_{\text{cell}}&=\left(\begin{array}{cc}{A}_{1}^{{\prime\:}}&\:{B}_{1}^{{\prime\:}}\\\:{C}_{1}^{{\prime\:}}&\:{D}_{1}^{{\prime\:}}\end{array}\right)\left(\begin{array}{cc}1&\:0\\\:{y}_{\text{D}\text{H}\text{R}}&\:1\end{array}\right)\left(\begin{array}{cc}{A}_{1}^{{\prime\:}}&\:{B}_{1}^{{\prime\:}}\\\:{C}_{1}^{{\prime\:}}&\:{D}_{1}^{{\prime\:}}\end{array}\right)\\&=\left(\begin{array}{cc}{M}_{\text{cell}}&\:{M}_{\text{cell}}\\\:{M}_{\text{cell}}&\:{M}_{\text{cell}}\end{array}\right),\end{aligned}$$

where $\:{A}_{1}^{{\prime\:}}={D}_{1}^{{\prime\:}}=\text{c}\text{o}\text{s}\left({k}_{1}{d}_{1}/2\right),{B}_{1}^{{\prime\:}}=\text{j}{z}_{1}\text{s}\text{i}\text{n}\left({k}_{1}{d}_{1}/2\right),{C}_{1}^{{\prime\:}}=\text{j}{y}_{1}\text{s}\text{i}\text{n}\left({k}_{1}{d}_{1}/2\right)$. The dispersion relation obtained by the TMM is given as:

27 $${\text{cos}}\left(\text{K}{d}_{1}\right)=\frac{1}{2}\left({M}_{\text{cell}}+{M}_{\text{cell}}\right)={\text{cos}}\left(k{d}_{1}\right)+\frac{1}{2}j{z}_{1}{y}_{DHR}{\text{sin}}\left(k{d}_{1}\right).$$

This equation can also be obtained by combining Eqs. S6 and S15 (Supporting Document). Hence, the dispersion relation obtained by the TMM and IRF method remains the same. For an N unit cell composed of DHRs, the transfer matrix of the system is:

28 $$\:{\text{M}}_{\text{c}\text{e}\text{l}\text{l}}^{\text{N}}=\left(\begin{array}{cc}{\text{M}}_{\text{c}\text{e}\text{l}\text{l}11}{\text{U}}_{\text{N}}-{\text{U}}_{\text{N}-1}&\:{\text{M}}_{\text{c}\text{e}\text{l}\text{l}12}{\text{U}}_{\text{N}}\\\:{\text{M}}_{\text{c}\text{e}\text{l}\text{l}21}{\text{U}}_{\text{N}}&\:{\text{M}}_{\text{c}\text{e}\text{l}\text{l}22}{\text{U}}_{\text{N}}-{\text{U}}_{\text{N}-1}\end{array}\right),$$

where $\:{U}_{N}=\text{sin}\left(N\text{K}{d}_{1}\right)/\text{sin}\left(\text{K}{d}_{1}\right)\:$is a Chebyshev polynomial of the second type $\:N$. In this case, the transmission coefficient can easy be deduced using the following equation:

29 $$\begin{aligned} T={\left|\frac{2}{{M}_{\text{cell}11}{U}_{N}-{U}_{N-1}+{y}_{1}{M}_{C\text{cell}12}{U}_{N}+{z}_{1}{M}_{\text{cell}21}{U}_{N}+\:{M}_{\text{cell}22}{U}_{N}-{U}_{N-1}}\right|}^{2}.\end{aligned}$$

### Defects inside periodic DHRs

**Fig. 5:**
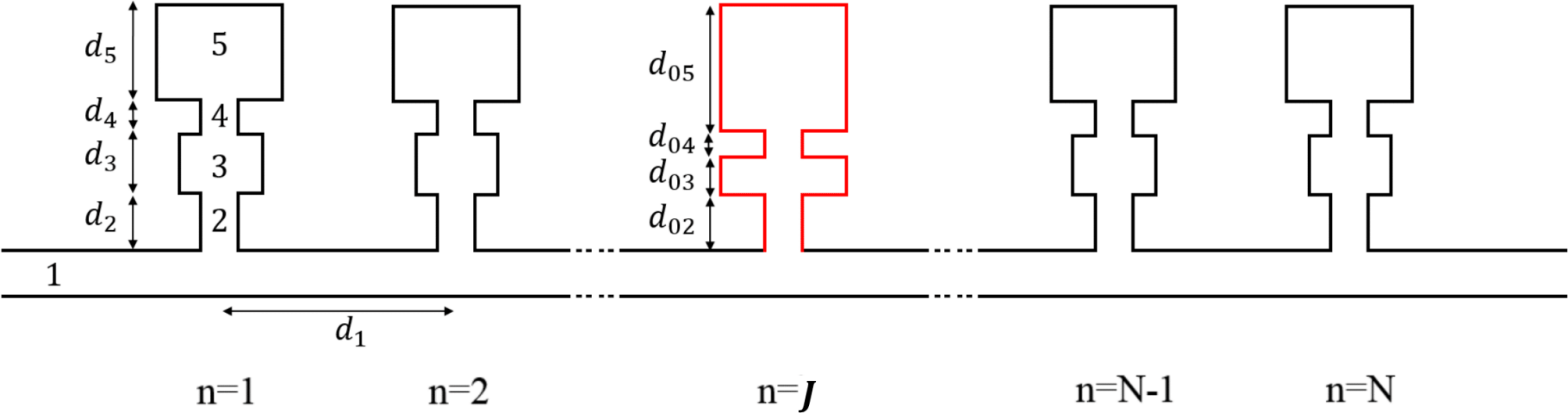
Illustration of finite periodic DHRs with defective DHR centrally located inside the structure. In this case, J represents the cell location in which the DHR is connected, and n represents the junctions where common DHRs are installed.

The presence of defect resonator in a finite periodic system formed by DHRs was investigated in this study. The defective DHR has different neck/cavity lengths compared with common resonators (Fig. 5).

The global matrix of a defective structure is given as:

30 $$\:M={\text{M}}_{\text{cell}}^{\text{n}}{M}_{\text{D}}{\text{M}}_{\text{cell}}^{n}.$$

Here, $\:N=2n+1$ denotes the total number of cells. $\:n$ is the unit cells at each side of defective cell. The defects are at the center of the structure. $\:{M}_{\text{D}}$ is the transfer matrix of the defective cell, as follows:

31 $$\:{M}_{\text{D}}=\left(\begin{array}{cc}{A}_{1}^{{\prime\:}}&\:{B}_{1}^{{\prime\:}}\\\:{C}_{1}^{{\prime\:}}&\:{D}_{1}^{{\prime\:}}\end{array}\right)\left(\begin{array}{cc}1&\:0\\\:{y}_{\text{D}}&\:1\end{array}\right)\left(\begin{array}{cc}{A}_{1}^{{\prime\:}}&\:{B}_{1}^{{\prime\:}}\\\:{C}_{1}^{{\prime\:}}&\:{D}_{1}^{{\prime\:}}\end{array}\right),$$

where $\:{y}_{\text{D}}$ is the admittance of the defective DHR.

## Numerical results

**Fig. 6:**
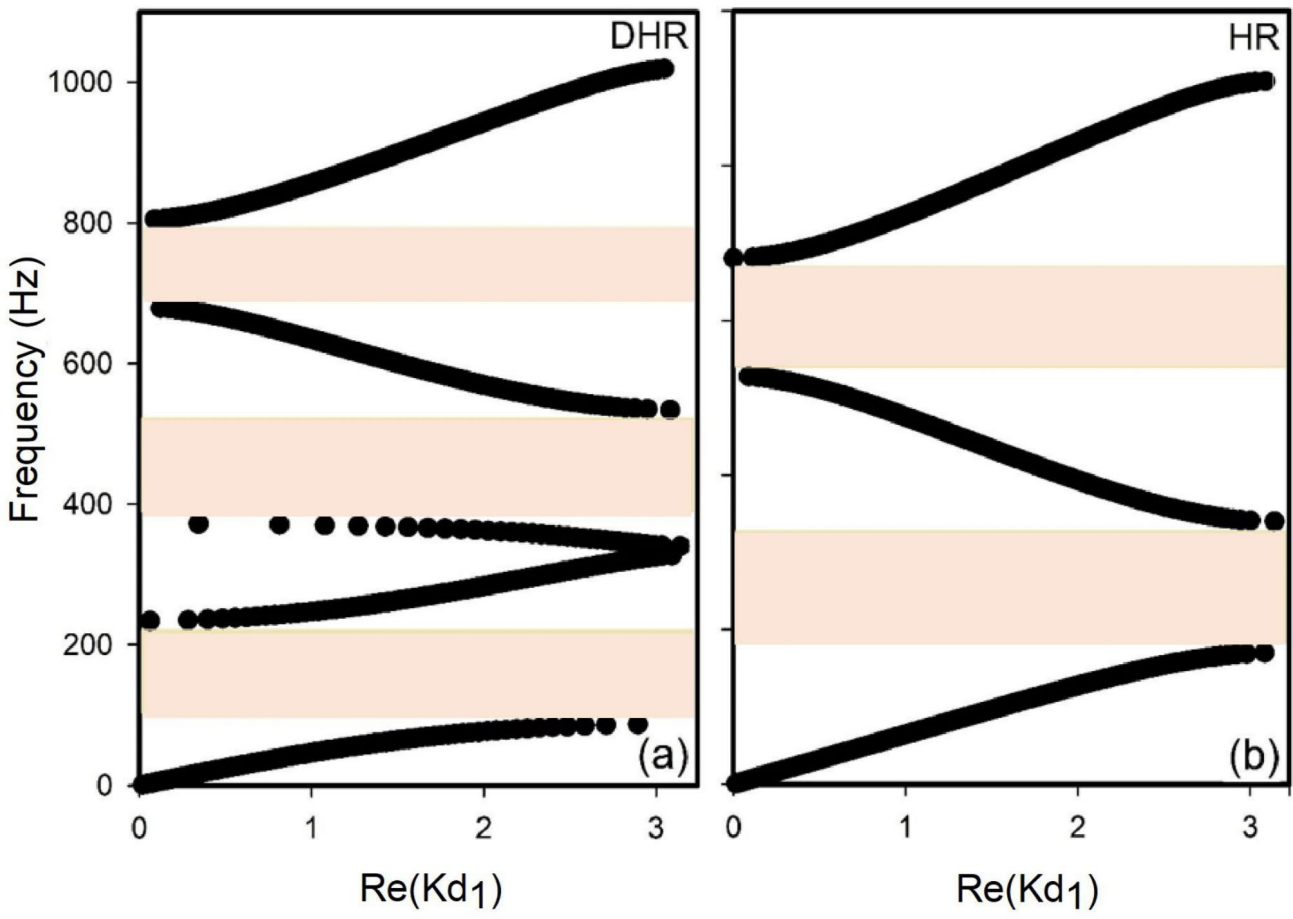
(**a**) Band structure of a perfect periodic structure formed by cylindrical DHRs $\:0<{\text{I}\text{m}(Kd}_{1})<\pi\:$ for $\:{d}_{1}$ = 50 cm,$\:\:{d}_{2}={d}_{4}=2$ cm, $\:{d}_{3}={d}_{5}=1$0.16 cm, $\:{s}_{1}$=5$\:\times\:$5 $\:{\text{c}\text{m}}^{2}$, $\:{r}_{2}={r}_{4}=$ 1.75 cm, $\:{r}_{3}={r}_{5}=$ 8.5 cm, $\:{r}_{i}(i=2-5)$ being the radius of the DHR elements. (**b**) Perfectly periodic band structure of simple HR (with only one resonator). These parameters were similar to those of DHR. The pink color represents the bandgap.

Figure 6 presents the simulation results depicting the band structure of periodic DHRs under the specific conditions $\:{d}_{2}={d}_{4},\: \text{ and}\:{d}_{3}={d}_{5}$. In addition to the resonance, interference of resonant frequencies occurs. The frequencies were 340 Hz and 680 Hz, respectively. The band structure showed three band gaps surrounded by four passbands. The 2nd band gap had the largest width (∆*f* = 162 Hz), while the 4th passband was the largest (∆*f* = 214 Hz). A comparison between the band structures of the periodic DHRs and simple HRs is presented in Fig. 6b. Initially, we examined the similarities between the bands for each structure. Clearly, the first passband of the HRs and that of the DHR are similar. The second and third passbands of the HR structure closely resembled the third and fourth passbands of the DHRs. Moreover, the 2nd band gap of the HRs structure is similar to the third DHRs band gap. Figure 6a clearly shows the effect of adding a second HR (in cascade). We observed the appearance of a new passband, specifically the second passband (Fig. 6a). The appearance of the latter decreased the width of the first passband (Fig. 6b) by half. The other bands were similar to those of the HR band, with a decrease in their widths.

One way to model the DHR’s dynamic behavior is as a mass-spring-damper system. The air within the necks acts as an oscillating mass, whereas a large volume of air serves as the spring element. Mass radiation was provided by fluid in the neck. The open end of the neck emits sound and provides another element of mass and radiation resistance. The wall of the necks provides additional strength, whereas the compression of the fluid in the cavities provides rigidity. Viscosity losses from the friction of the oscillating air in the neck and radiation losses at the neck’s ends are two symptoms of damping. However, these effects were not considered in this study. Hence, with reference to acoustic applications, a DHR exhibits two resonance frequencies. On contrary, a single HR exhibits only one resonant frequency^11,43,44^.

**Fig. 7:**
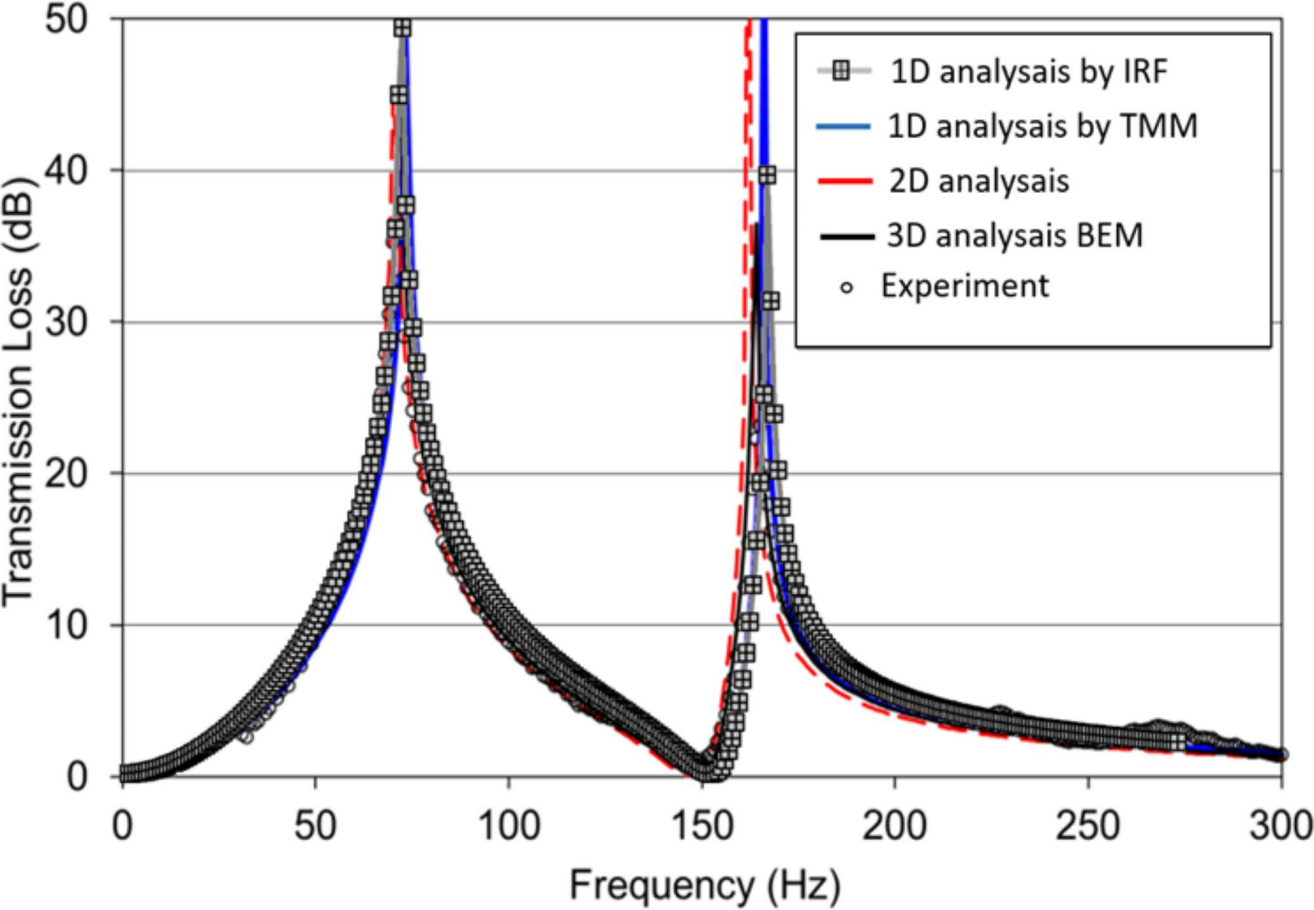
The TL of the DHR without mean flow. Comparison of the 1D analytical IRF predictions (gray line with square, crossed), TMM (blue line)^19^, 2D (red line) and 3D (black line) predictions by boundary element method (BEM) (red and black lines) vs. the experiment (circle symbols)^19,20^.

**Table 1:** Geometric and physical parameters of the DHR aligning with^19,20^.

Parameter	Value
First neck length $\:{d}_{2}$ (cm)	8.5
Second neck length $\:{d}_{4}$ (cm)	7.62
First cavity length $\:{d}_{3}$ (cm)	20.32
Second cavity length $\:{d}_{5}$ (cm)	10.16
First neck radius $\:{r}_{2}$ (cm)	$\:2$
Second neck radius $\:\:{r}_{4}$ (cm)	1.75
First cavity radius $\:{r}_{3}$ (cm)	7.62
Second cavity radius $\:\:{r}_{5}$ (cm)	7.62
Duct cavity cross-section $\:{s}_{1}$ (cm^2^)	18.49
*N*	11
$N^{\prime}$	1
$\:{\rho\:}_{0}\:({\text{kg/m}}^{3})$	$\:1.2$
$\:{c}_{0}\:{\text{(m/s)}}$	$\:343$

**Fig. 8:**
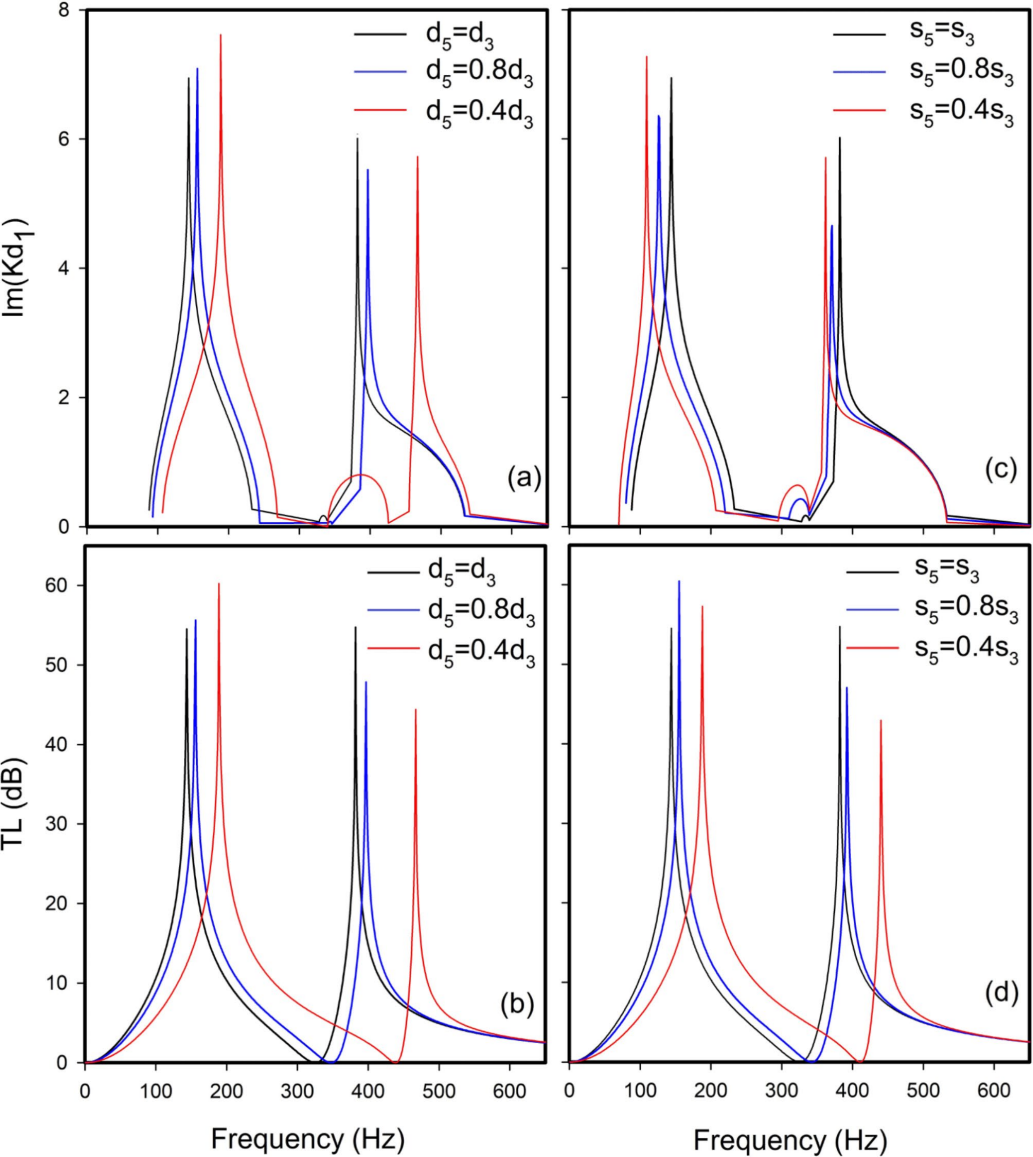
Acoustic characteristics of (**a**) $\:\text{I}\text{m}\left({Kd}_{1}\right)$, (**b**) TL of a DHR for different values of $\:{d}_{5}$. Acoustic characteristics of (**c**) $\:\text{I}\text{m}\left({Kd}_{1}\right)$, (**d**) TL of a DHR for different values of $\:{S}_{5}$, in the absence of flow.

The DHR configuration in Fig. 5 is employed in the subsequent numerical simulations. The cross-sectional area of the primary duct is $\:4.3\:\text{c}\text{m}\times\:4.3\:\text{c}\text{m}$ and is connected to DHRs. In Fig. 7, our simulation results (represented by gray square crossed symbols) for the transmission are compared with the IRF, 1D TMM^19^, 2D/3D analysis by the boundary element method (BEM)^19,20^, as well as the experiment data^19,20^ from published sources for a specific geometrical configuration (Table 1). The results show good agreement across all methods. Then, TL (Eq. 25) is studied, in the absence of flow, by variations in the length and cross-section of the DHR second cavity. Figure 8b, d illustrate how variations in $\:{d}_{5}$ and $\:{s}_{5}$ can impact the TL. Several theoretical and applied studies have been conducted to determine the dimensions of the DHR^21,41,45,46^. Attenuating sound of medium and low frequencies is a common application of HR.

Clearly, decreasing $\:{d}_{5}$ shifts both peaks to higher frequencies. Altering the cross-sectional area of the second cavity $\:{s}_{5}$ (Fig. 8b) also shifts both peaks towards higher frequencies. Notably, in Fig. 8b, the TL intensity of the first peak increases for values below $\:{d}_{3}$, the second resonance peak shows an opposite behavior, consistent with Fig. 8b. These findings lead to the conclusion that the first TL peak has a significant impact, especially as resonant frequencies approach lower frequencies, correlating with increased losses. Moreover, decreasing the lengths or cross-sectional areas of DHR cavities shifts resonance peaks to higher frequencies.

Additionally, $\:{\text{I}\text{m}(\text{K}\text{d}}_{1})$ describes evanescent waves within band gaps, aligns closely with resonant frequencies in Fig. 8a, except for Fig. 8c, where the peak slightly shifts to lower frequencies. Comparing our study with^43^, optimal attenuation depends on the engineer’s objectives. For instance, in constrained spaces requiring optimal attenuation, designs like Seo’s are advantageous. Conversely, DHRs are preferable when multiple high attenuations (two in the case of DHRs) are necessary without space limitations. A simple HR can only attenuate a single harmonic pulsation, while hydraulic flow pulsations typically involve multiple higher harmonics in addition to the fundamental frequency^47^. DHRs are thus highly suitable for attenuating multiple hydraulic flow pulsations effectively.

**Fig. 9:**
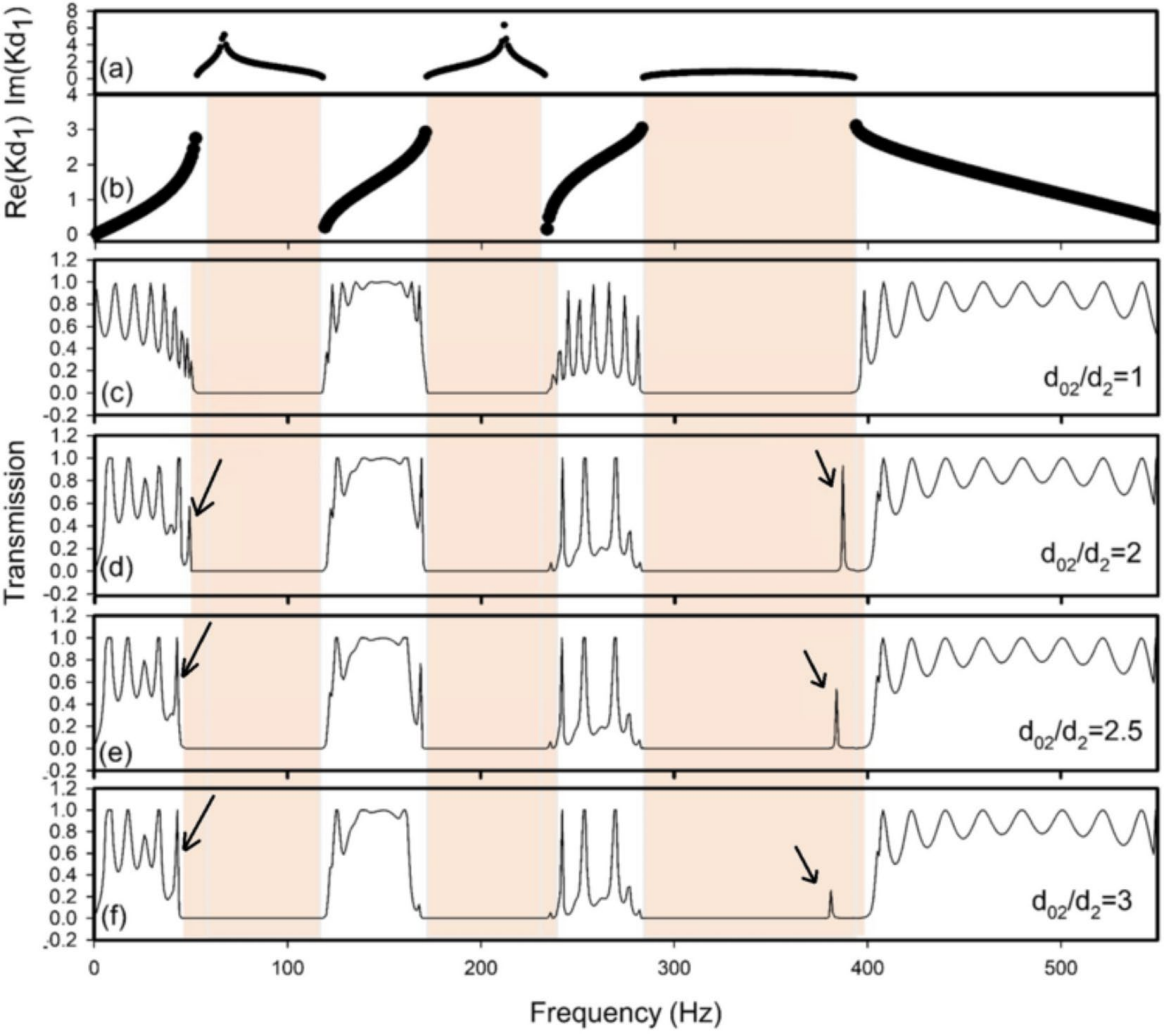
Transmission coefficient versus the frequency at different lengths of first neck d_02_ of the defective DHR. The defect is located in the cell $\:n\:=\:6$, for $\:N\:=\:11$, $\:{d}_{1}=50$ cm, $\:{d}_{2}=4.4$ cm, $\:{d}_{3}={d}_{03}=10.16$ cm, $\:{d}_{4}={d}_{04}=7.62$ cm, $\:{d}_{5}={d}_{05}=20.30$ cm, $\:{s}_{1}=$5$\:\times\:$5$\:\:{\text{c}\text{m}}^{2}$, $\:{r}_{2}={r}_{02}=1.75$ cm, $\:{r}_{3}={r}_{03}=8.5$ cm, $\:{r}_{4}={r}_{04}={r}_{03}$, $\:{r}_{5}={r}_{05}={r}_{3}.$ (**a**) $\:\text{I}\text{m}\left({Kd}_{1}\right)$ at $\:{d}_{02}/{d}_{2}=1$, (**b**) $\:\text{r}\text{e}\text{a}\text{l}\left({Kd}_{1}\right)$ at $\:{d}_{02}/{d}_{2}=1$, (**c**) transmission at $\:{d}_{02}/{d}_{2}=1$, (**d**) transmission at $\:{d}_{02}/{d}_{2}=2$, (**e**) transmission at $\:{d}_{02}/{d}_{2}=2.5$, and (**f**) transmission at $\:{d}_{02}/{d}_{2}=3$.

As seen in Fig. 9a, the imaginary component of the dimensionless variable $\:{Kd}_{1}$ characterizes the amplitude decay of waves evanescent within these band gaps. As the number of cells (N) rises, the behavior of the cells resembles the infinite structure seen in Fig. 9b, indicating the appearance of band gaps. The transmission of sound waves through eleven identical DHR cells is represented in Fig. 9c. The periodic nature of the system has a major role in the creation of these band gaps. Sound waves inside these band gaps are significantly attenuated by DHRs due to multiple Brag scattering.

The transmission coefficient of the defective construction is clear in Fig. 9d–f as a function of the defective neck length ($\:{d}_{02}$). The other parameters are the same as for regular resonators. The flaw is situated at the periodic structure’s core, where air is thought to be present in both the main tube and the resonators. The confined mode is created within the first band gap by introducing a defective DHR inside the structure. By raising $\:{d}_{02}$, the peak is red-shifted and goes out of the band gap. The third band gap shows that this pattern continues, with the confined mode continuously moving down to lower frequencies. The transmission spectrum is near to zero by increasing $\:{d}_{02}/{d}_{2}$, which is similar to the properties of a high-pass filter. The flaw can cause the filter type to be entirely altered. Furthermore, reducing the first neck’s length lowers the transmission of acoustic waves at the third pass band. The defect mode transmission, located at the third band gap, can be enhanced by a second dimensional defect in the same defective DHR.

**Fig. 10:**
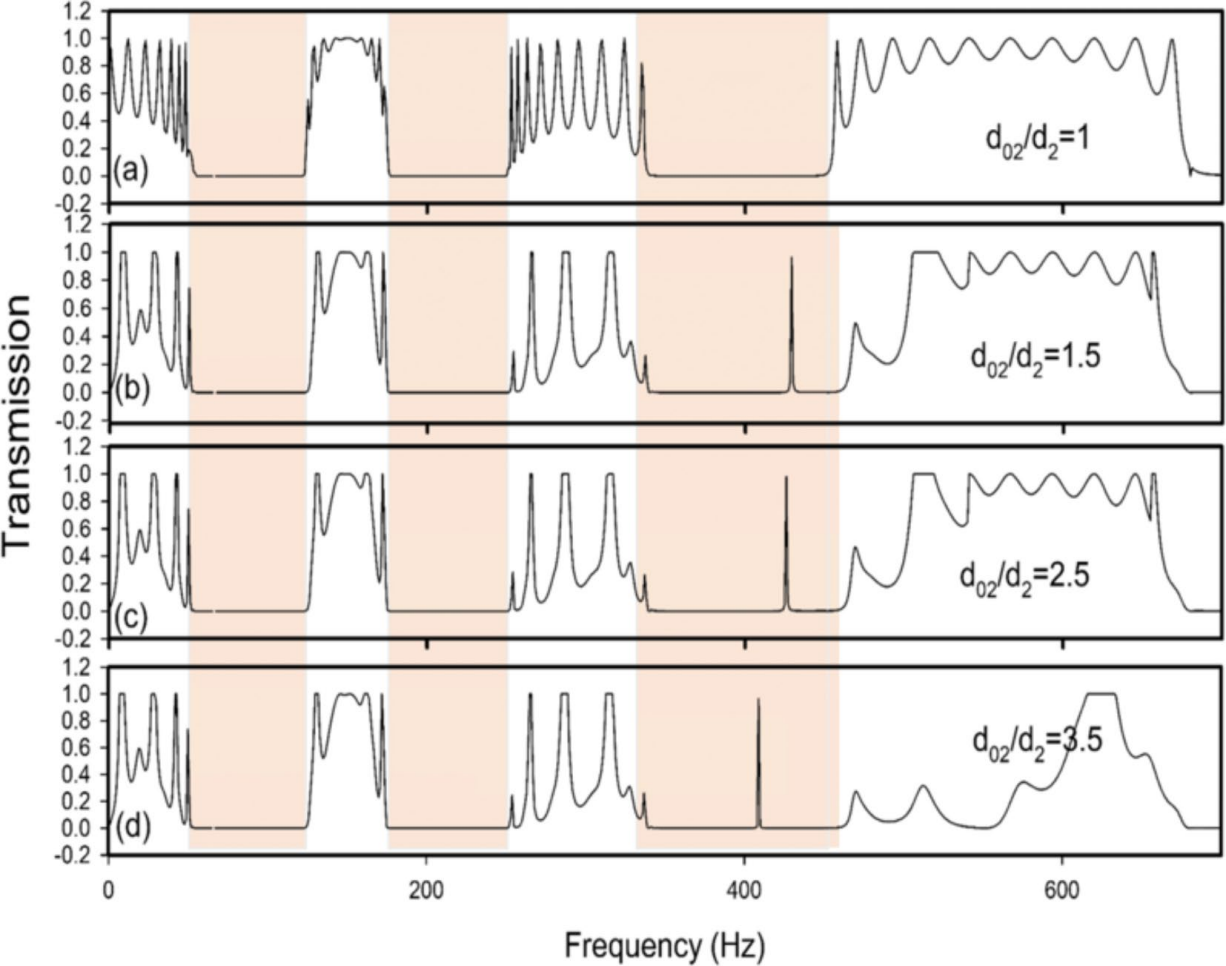
Transmission coefficient versus the frequency for different values of the defective length $\:{d}_{02}$ of the first neck for 11 identical cells ($\:n=6\:$and$\:\:N=11$) filled with the air. The second defect is located in the first cavity $\:{d}_{03}$=$\:{2d}_{3}$ of the same defective DHR (*n* = 6). The other parameters are similar to those in Fig. 9. (**a**) at $\:{d}_{02}/{d}_{2}=1$ (without defect), (**b**) at $\:{d}_{02}/{d}_{2}=1.5$, (**c**) at $\:{d}_{02}/{d}_{2}=2.5$, and (**d**) at $\:{d}_{02}/{d}_{2}=3.5$.

Figure 10a–d analyze the case where $\:{d}_{03}=2{d}_{3}$ and the length of the first neck of the defective DHR varies. Moreover, the pass bands are significantly impacted by the change of both $\:{d}_{02}$ and the inadequate cavity length $\:{d}_{03}$. It is clear that the latter affects the migration of the localized strong transmission mode towards lower frequencies. A defect’s effect on pass-band transmission is substantial when it exists in a cavity.

**Fig. 11:**
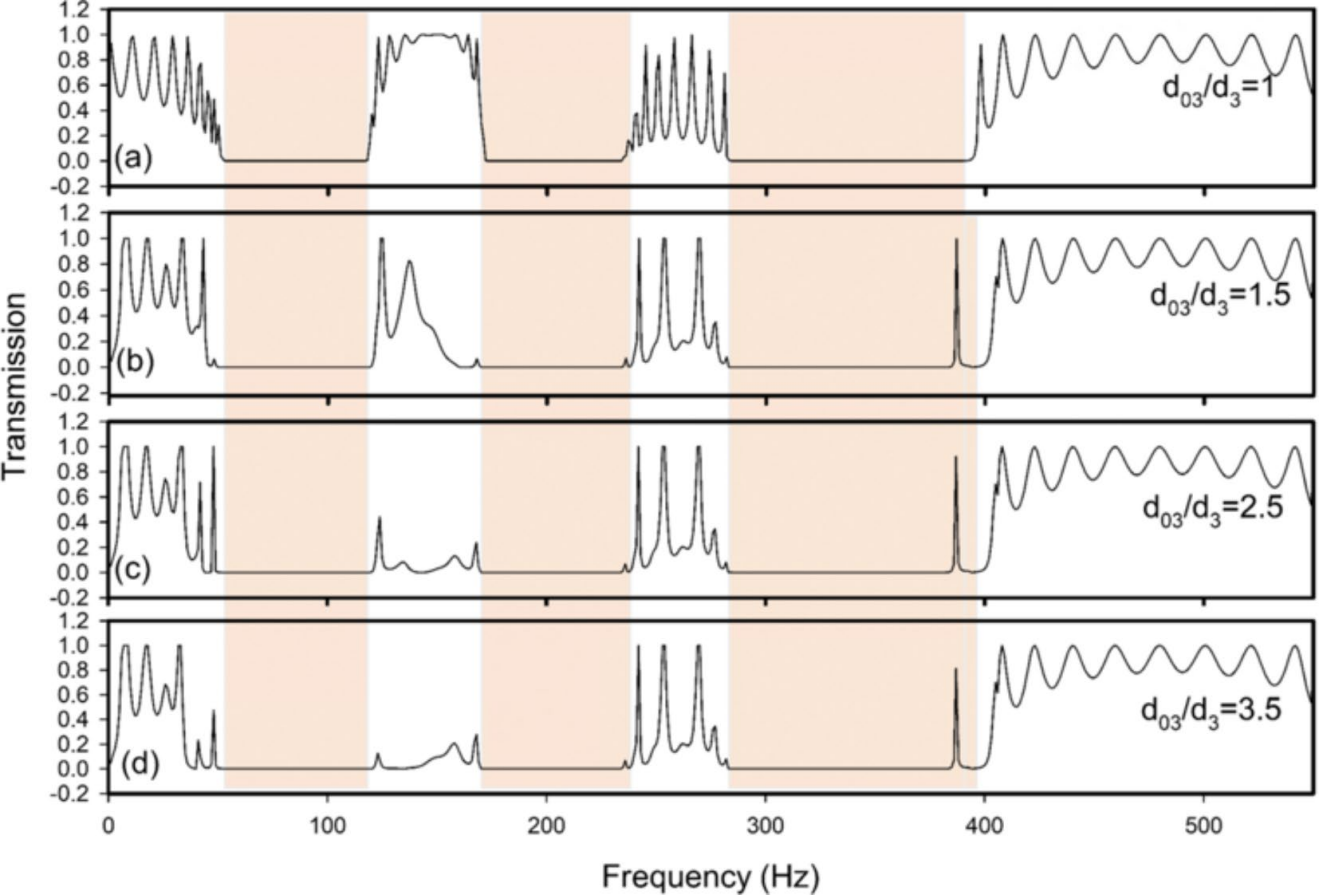
Variation of the transmission coefficient as a function the frequency for different defective cavity length ($\:{d}_{03}$) in the case where $\:{d}_{02}={2d}_{2}$. The other parameters are similar to those shown in Fig. 9. (**a**) at $\:{d}_{03}/{d}_{3}=1$ (without defect), (**b**) at $\:{d}_{03}/{d}_{3}=1.5$, (**c**) at $\:{d}_{03}/{d}_{3}=2.5$, and (**d**) at $\:{d}_{03}/{d}_{3}=3.5$.

The impact of first length change of the cavity defect is depicted in Fig. 11a–d. Strong attenuations of the waves within the second pass-band can be obtained in this case due to the greater value of $\:{d}_{03}$. When $\:{d}_{02}=3.5{\:d}_{2}$, the second pass-band practically vanishes. Defect modes’ mutual interactions have a significant impact on the second pass-band. We adjusted the first resonator’s neck defect in Fig. 11 on the same value ($\:{d}_{02}=2{d}_{2}$) and the defective cavity ($\:{d}_{03}$) is lengthened because the case of $\:{d}_{02}=2{d}_{2}$ in Fig. 9 offers the largest localized mode inside the third bang gap. The second pass-band is impacted by increased reflections within the first cavity. In fact, the mutual interaction of the resonance frequencies caused by the two defects ($\:{d}_{02}$ and $\:{d}_{03}$) in the second pass-band which attenuate an important part of it. The existence of a defect in the first cavity makes the localized mode keep the same frequency position 387 Hz. While its transmission decreases for $\:{d}_{03}>{d}_{3}$, it is also a way to show the important effect played by the defective cavity. The coupling effect of peaks in this case decreases the transmission of defect mode. Thus, by introducing two defects we are able to:

(i) control the transmission inside pass-band and even change a pass-band to a band gap.
(ii) control the transmission of the defect mode inside band gaps.

Indeed, the two defects ($\:{d}_{02}$ and $\:{d}_{03}$) mutually interact to create resonance frequencies in the second pass-band, which attenuates a significant portion of it. The confined mode maintains its 387 Hz frequency location due to a defect in the first cavity. Even though its transmission drops for $\:{d}_{03}>{d}_{3}.$ Nevertheless, it serves as a useful illustration of the significant role the defective cavity played. In this instance, the coupling effect of the defect modes reduces the defect mode’s transmission. Therefore, we may:

(i) regulate the transmission inside the pass-band and even convert a pass-band into a band gap by adding two defects.
(ii) control the transmission of the defect mode inside the band gaps.

## Conclusion

This paper investigates noise reduction techniques using periodic DHRs with dimensional defects in the center of the structure. We generate broad acoustic band gaps appropriate for acoustic filtering by utilizing periodicity. The DHR has two unique resonance frequencies, according to simulation data which were in good agreement, particularly at lower frequencies, with the IRF predictions and assessments from the BEM, TMM, as well as the experimental data from literature. Optimizing noise reduction is the goal of aligning interference of resonant frequencies. By comparing to single resonators, this method promises efficient reduction of wideband noise, especially at lower frequencies. Because of its ease of use, the TMM makes it possible to expand the study to N′ cascaded resonators, which improved noise reduction even further. The defects under investigation are situated within the same DHR, specifically at the first neck and/or cavity. This work presents a practical method to improve the transmission of defect modes by creating a secondary dimensional defect in the same DHR. This enhancement results from mutual interaction between resonant modes induced by both defects. Such interaction can significantly attenuate sound within the pass band, transforming the defective structure into a selective filter.

## Electronic supplementary material

Below is the link to the electronic supplementary material.

Supplementary Material 1

## Data Availability

Requests for materials or code should be addressed to Zaky A. Zaky and M. El Malki.
