# Supplementary material for: Noise filter using a periodic system of dual Helmholtz resonators
Source: Sci Rep. 2024 Oct 23;14:24987. doi: 10.1038/s41598-024-74799-2 (PMC11499606; doi:10.1038/s41598-024-74799-2)
Supplement: Supplementary file 1 — Supplementary Material 1 [file 41598_2024_74799_MOESM1_ESM.docx]

Supplementary Material: Noise Filter Using a Periodic System of Dual Helmholtz Resonators

Mohamed El Malki ^1^, Ali Khettabi ^1^, Mohammed Sallah ^2^, Zaky A. Zaky ^3, 4, 5,^*

^1^ Laboratory of Materials, Waves, Energy and Environment, Department of Physics, Faculty of Sciences, Mohammed First University, Oujda 60000, Morocco

^2^ Department of Physics, College of Sciences, University of Bisha, P.O. Box 344, Bisha 61922, Saudi Arabia.

^3^ TH-PPM Group, Physics Department, Faculty of Science, Beni-Suef University, Beni Suef 62514, Egypt.

^4^ Academy of Scientific Research and Technology (ASRT), Cairo, Egypt

^5^ Frank Laboratory of Neutron Physics, Joint Institute for Nuclear Research, 141980, Dubna, Russia

***** Correspondence: [zaky.a.zaky@science.bsu.edu.eg](mailto:zaky.a.zaky@science.bsu.edu.eg)

# IRF of the DHR

For a unit cell, the Green’s function of DHR is defined by

| $g_{\mathrm{DHR}}^{-1}\left( M,M \right)=\left( \begin{matrix} A_{2} & B_{2} & 0 & 0 & 0 \\ B_{2} & C_{1} & B_{3} & 0 & 0 \\ 0 & B_{3} & C_{2} & B_{4} & 0 \\ 0 & 0 & B_{4} & C_{3} & B_{5} \\ 0 & 0 & 0 & B_{5} & A_{5} \end{matrix} \right) ,$ | (S1) |
| --- | --- |

$w$here${{C_{1}=A}_{2}+A_{3}, C_{2}{=A}_{3}+A_{4}, {C_{3}=A}_{4}+A_{5}, A}_{i}=-F_{i}C_{i}/S_{i}, B_{i}=F_{i}/S_{i}, (i=2-5), F_{i}=-j\omega/z_{i}, (j=\sqrt{-1}), C_{i}=\cosh\left( kd_{i} \right)$, $S_{i}=\sinh\left( kd_{i} \right),k=\omega/c_{0}.$ $g_{\mathrm{DHR}}^{-1}(M,M)$ is a block matrix, each block represent the elements of the Green's functions of one of the four finite waveguides constituting the DHR. While the adjugated matrix $\mathrm{adj}(g_{\mathrm{DHR}}^{-1}(M,M))$ is giving by:

| $\mathrm{adj} \left( g_{\mathrm{DHR}}^{-1}\left( M,M \right) \right)=\left( \begin{matrix} A^{'} & B^{'} & C^{'} & D^{'} & E^{'} \\ F^{'} & G^{'} & H^{'} & I^{'} & J^{'} \\ K^{'} & L^{'} & M^{'} & N^{'} & O^{'} \\ P^{'} & Q^{'} & R^{'} & S^{'} & T^{'} \\ U^{'} & V^{'} & W^{'} & X^{'} & Y^{'} \end{matrix} \right).$ | (S2) |
| --- | --- |

Elements of $\mathrm{adj} \left( g_{\mathrm{DHR}}^{-1}\left( M,M \right) \right)$ are numerically calculated. At the reduced interface space $M= ADDIN EN.CITE <EndNote><Cite ExcludeYear="1"><Author>https://www.who.int/malaria/</Author><RecNum>515</RecNum><record><rec-number>515</rec-number><foreign-keys><key app="EN" db-id="2xf0dsartse59getaz65rrawxazafp9pr92p" timestamp="1624915026">515</key></foreign-keys><ref-type name="Journal Article">17</ref-type><contributors><authors><author>WHO report 2019 available at https://www.who.int/malaria/</author><author>publications/world-malaria-report-2019/en/</author></authors></contributors><titles></titles><dates></dates><urls></urls></record></Cite></EndNote>\{0\}$. representing the junction between the connected DHR in the waveguide, and by using Eqs. S1 and S2, $g_{\mathrm{DHR}}(0,0)$ is written as:

| $g_{\mathrm{DHR}}\left( 0,0 \right)=\frac{A_{\mathrm{DHR}}}{B_{\mathrm{DHR}}}.$ | (S3) |
| --- | --- |

In Eq. S2, only the quantity *A'* is needed, which is the first element of the matrix $\mathrm{adj} \left( g_{\mathrm{DHR}}^{-1}\left( M,M \right) \right)$. Hence,

| $A_{\mathrm{DHR}}=A'$, | (S4) |
| --- | --- |
| $B_{\mathrm{DHR}}=\det\left[ g_{\mathrm{DHR}}^{-1}(M,M) \right]$  $=A_{2}\left\vert\begin{matrix} A_{2}+A_{3} & B_{3} & 0 & 0 \\ B_{3} & A_{3}+A_{4} & B_{4} & 0 \\ 0 & B_{4} & A_{4}+A_{5} & B_{5} \\ 0 & 0 & B_{5} & A_{5} \end{matrix} \right\vert-B_{2}\left\vert\begin{matrix} B_{2} & B_{3} & 0 & 0 \\ 0 & A_{3}+A_{4} & B_{4} & 0 \\ 0 & B_{4} & A_{4}+A_{5} & B_{5} \\ 0 & 0 & B_{5} & A_{5} \end{matrix} \right\vert$. | (S5) |

Finally, we derive the Green's function element of the DHR within the reduced interface space.

| $g_{\mathrm{DHR}}^{-1}(0,0)=C_{\mathrm{DHR}}/D_{\mathrm{DHR}},$ | (S6) |
| --- | --- |

where,

| $\begin{aligned} C_{\mathrm{DHR}}=&j\omega\left[ y_{2}\tan\left( kd_{2} \right)+y_{3}\tan\left( kd_{3} \right)+y_{4}\tan\left( kd_{4} \right)+y_{5}\tan\left( kd_{5} \right) \right. \\ &-y_{2}z_{3}y_{5}\tan\left( kd_{2} \right)\tan\left( kd_{3} \right)\tan\left( kd_{5} \right)-y_{2}z_{4}y_{5}\tan\left( kd_{2} \right) \\ &\left. \tan\left( kd_{4} \right)\tan\left( kd_{5} \right)-y_{3}z_{4}y_{5}\tan\left( kd_{3} \right)\tan\left( kd_{4} \right)\tan\left( kd_{5} \right) \right], \end{aligned}$ | (S7) |
| --- | --- |
| $\begin{aligned} D_{\mathrm{DHR}}=&1-z_{4}y_{5}\tan\left( kd_{4} \right)\tan\left( kd_{5} \right)-z_{3}y_{4}\tan\left( kd_{3} \right)\tan\left( kd_{4} \right)-z_{3}y_{5} \\ &\tan\left( kd_{3} \right)\tan\left( kd_{5} \right)-z_{2}y_{3}\tan\left( kd_{2} \right)\tan\left( kd_{3} \right)-z_{2}y_{5}\tan\left( kd_{2} \right) \\ &\tan\left( kd_{5} \right)-z_{2}y_{4}\tan\left( kd_{2} \right)\tan\left( kd_{4} \right)+z_{2}y_{3}z_{4}y_{5}\tan\left( kd_{2} \right) \\ &\tan\left( kd_{3} \right)\tan\left( kd_{4} \right)\tan\left( kd_{5} \right). \end{aligned}$ | (S8) |

If we consider the special case where $d_{2}=d_{4}\text{, and }d_{3}=d_{5}$, Eqs. S7 and S8 become [2]:

| $\begin{aligned} C_{\mathrm{DHR}}=&j\omega\left[ 2\left( y_{2}\tan\left( kd_{2} \right)-y_{3}\tan\left( kd_{3} \right) \right)-\left( y_{3}+z_{2}y_{2}^{2} \right)\tan^{2}\left( kd_{2} \right) \right. \\ &\left. \tan\left( kd_{3} \right)-\left( y_{2}+z_{2}y_{3}^{2} \right)\tan\left( kd_{2} \right)\tan^{2}\left( kd_{3} \right) \right] \end{aligned}$ | (S9) |
| --- | --- |
| $\begin{aligned} D_{\mathrm{DHR}}=&1-\left( 3z_{2}y_{3}+z_{3}y_{2}-z_{2}^{2}y_{3}^{2}\tan\left( kd_{2} \right)\tan\left( kd_{3} \right) \right)\tan\left( kd_{2} \right) \\ &\tan\left( kd_{3} \right)-\tan^{2}\left( kd_{2} \right)-\tan^{2}\left( kd_{3} \right) \end{aligned}$ | (S10) |

Using the TMM [58] (Eq. 21 in manuscript), we can deduce the relation that links the inverse of the Green’s function of the DHR and its acoustic impedance of the DHR ($z_{\mathrm{DHR}}$), which is given by:

| $g_{\mathrm{DHR}}^{-1}(0,0)=\omega/z_{\mathrm{DHR}}$. | (S11) |
| --- | --- |

## Dispersion relation of the infinite DHRs

The infinite DHRs are composed on a periodic DHR. Thus, the inverse of the Green’s function of the elementary cell is given by:

| $g^{-1}\left( 0,d_{1} \right)=\left( \begin{matrix} A_{1}+g_{DHR}^{-1}(0,0) & B_{1} \\ B_{1} & A_{1} \end{matrix} \right)$. | (S12) |
| --- | --- |

The Green's function of the overall system is derived as an infinite matrix, composed by combining the elements $g_{\mathrm{DHR}}^{-1}(0,0)$, which are defined within the interface domain of each site or junction.

| $g_{DHR\infty}^{-1}\left( M,M \right)=\left( \begin{matrix} \ddots& \ddots& \ddots& & & & \\ & B_{1} & A_{1}+A_{2} & B_{1} & & & \\ & & B_{1} & A_{1}+A_{2} & B_{1} & & \\ & & & B_{1} & A_{1}+A_{2} & B_{1} & \\ & & & & \ddots& \ddots& \ddots\end{matrix} \right),$ | (S13) |
| --- | --- |

where ${A=-2A_{1}+g}_{\mathrm{DHR}}\left( 0,0 \right),$ and $B_{1}=F_{1}/S_{1}$. The matrix $g_{\infty}^{-1}(M,M)$ is equivalent to a dynamic matrix of a monoatomic linear chain with a one-spring constant. By analogy with the latter, the dispersion relation of the infinite DHR structure is given by:

| $2A_{1}+g_{\mathrm{DHR}}^{-1}(0,0)+B_{1}\left( e^{\mathrm{jK}d_{1}}+e^{-\mathrm{jK}d_{1}} \right)=0,$ | (S14) |
| --- | --- |

K is the Bloch wave number. Thus,

| $\cos\left( \mathbf{K}d_{1} \right)=\xi=C_{1}-\frac{1}{2}\frac{S_{1}}{F_{1}}g_{\mathrm{DHR}}^{-1}(0,0)$, | (S15) |
| --- | --- |

$g_{\mathrm{DHR}}^{-1}(0,0)$ is given by Eq. S6. In case of presence of a defect inside the structure, the frequencies corresponding to the localized peaks of the infinite structure can be expressed as detailed in [3, 4] by:

| $1+\frac{\gamma t}{\left( t^{2}-1 \right)}=0$. | (S16) |
| --- | --- |

The quantity $t$ given as $t+\frac{1}{t}=2\xi$ is a very important quantity associated with the development of the band structure in a periodic medium. It is given by:

| $t=e^{\left( j\mathbf{K}d_{1} \right)}=\left( \begin{matrix} \xi-\left( \xi^{2}-1 \right)^{\frac{1}{2}} & \text{ for } & \xi\succ1 \\ \xi+\left( \xi^{2}-1 \right)^{\frac{1}{2}} & \text{ for } & \xi\prec-1 \\ \xi+j\left( 1-\xi^{2} \right)^{\frac{1}{2}} & \text{ for } & -1\prec\xi\prec1 \end{matrix} \right.$ , | (S17) |
| --- | --- |

$w$hile $\gamma$ represents the perturbation due to the defect insertion inside the structure, and it is given by:

| $\gamma=\frac{S_{1}}{F_{1}}\left[ g_{\mathrm{DHR}_{0}}^{-1}(0,0)-g_{\mathrm{DHR}}^{-1}(0,0) \right]$, | (S18) |
| --- | --- |

$g_{\mathrm{DHR}}^{-1}(0,0)$ being the inverse of the Green’s function of a common DHR, while ${g_{\mathrm{DHR}}^{-1}}_{0}(0,0)$ is that of the defective DHR given by:

| $g_{\mathrm{DHR}_{0}}^{-1}(0,0)=\omega/z_{\mathrm{DHR}_{0}}$, | (S19) |
| --- | --- |

${z_{\mathrm{DHR}}}_{0}$ being acoustic impedance of defective DHR:

| $z_{\mathrm{DHR}_{0}}=j\omega\rho_{0}d_{02}\frac{\left( -\omega^{2}/\omega_{03}^{2}+1 \right)\left( -\omega^{2}/\omega_{05}^{2}+1 \right)-R_{0}}{\left( -\omega^{2}/\omega_{03}^{2}+1 \right)\left( -\omega^{2}/\omega_{05}^{2}+R \right)-R_{0}}$ , | (S20) |
| --- | --- |

where$R_{0}=\left( s_{04}/s_{02} \right)/\left( d_{04}/d_{02}+s_{04}/s_{02} \right)$, $\omega_{03}= \sqrt{c_{0}^{2}\left( s_{02}/d_{02}+s_{04}/d_{04} \right)/V_{04}}$, $\omega_{05}= \sqrt{c_{0}^{2}s_{04}/d_{04}V_{04}}$ , $V_{0i}, d_{0i},$ and $s_{0i}$are respectively volumes of defective DHR elements (necks and cavities), their lengths and cross-sections $(i=2-5)$. In presence of a defect, the transmission coefficient is given as:

| $T=\frac{2F_{1}A_{12}}{\left( A_{11}-F_{1} \right)\left( A_{22}-F_{1} \right)-A_{21}A_{12}}$, | (S21) |
| --- | --- |

where $A_{11}, A_{12}, A_{21}, A_{22}$ are the elements of the defective matrix. Thus, Eq. S22 can be written as [4]:

| $T=\left\vert\frac{2\sin\left( k_{1}d_{1} \right)\left( t^{2}-1 \right)t^{N}}{\left( \alpha_{1}^{2}-\alpha_{2}^{2}t^{2N} \right)\left( t^{2}-1 \right)+\eta\gamma} \right\vert^{2}$, | (S22) |
| --- | --- |

$w$here:

| $\eta=t\left[ \alpha_{1}^{2}+\alpha_{2}^{2}t^{2N} \right]-\alpha_{1}\alpha_{2}\left[ t^{2\left( N-J+1 \right)}+t^{2n} \right],\left( 1<J<N \right),$ | (S23) |
| --- | --- |
| $\alpha_{1}=1-te^{\left( jk_{1}d_{1} \right)}$ | (S24) |
| $\alpha_{2}=t-e^{\left( jk_{1}d_{1} \right)}$ | (S25) |

The integer *J* in Eq. S23 represents the site, which corresponds to the position of the defect inside the finite structure.

# References

[1] W. r. a. a. <https://www.who.int/malaria/> and publications/world-malaria-report-2019/en/.

[2] T. Cox and P. d’Antonio, *Acoustic absorbers and diffusers: theory, design and application*: CRC press, 2016.

[3] H. Al-Wahsh, A. Akjouj, B. Djafari-Rouhani, J. O. Vasseur, L. Dobrzynski, and P. A. Deymier, "Large magnonic band gaps and defect modes in one-dimensional comblike structures," *Physical Review B,* vol. 59, p. 8709, 1999. <https://doi.org/10.1103/PhysRevB.59.8709>

[4] J. O. Vasseur, A. Akjouj, L. Dobrzynski, B. Djafari-Rouhani, and E. El Boudouti, "Photon, electron, magnon, phonon and plasmon mono-mode circuits," *Surface science reports,* vol. 54, pp. 1-156, 2004. <https://doi.org/10.1016/j.surfrep.2004.04.001>
